# Evidence-based practice profiles of physiotherapists transitioning into the workforce: a study of two cohorts

**DOI:** 10.1186/1472-6920-11-100

**Published:** 2011-11-29

**Authors:** Maureen P McEvoy, Marie T Williams, Timothy S Olds, Lucy K Lewis, John Petkov

**Affiliations:** 1 School of Health Sciences, University of South Australia, North Tce, Adelaide, 5000, South Australia; 2 Centre of Regional Engagement, University of South Australia, Mt Gambier campus, Mt Gambier, 5290, South Australia

## Abstract

**Background:**

Training in the five steps of evidence-based practice (EBP) has been recommended for inclusion in entry-level health professional training. The effectiveness of EBP education has been explored predominantly in the medical and nursing professions and more commonly in post-graduate than entry-level students. Few studies have investigated longitudinal changes in EBP attitudes and behaviours. This study aimed to assess the changes in EBP knowledge, attitudes and behaviours in entry-level physiotherapy students transitioning into the workforce.

**Methods:**

A prospective, observational, longitudinal design was used, with two cohorts. From 2008, 29 participants were tested in their final year in a physiotherapy program, and after the first and second workforce years. From 2009, 76 participants were tested in their final entry-level and first workforce years. Participants completed an Evidence-Based Practice Profile questionnaire (EBP^2^), which includes self-report EBP domains [Relevance, Terminology (knowledge of EBP concepts), Confidence, Practice (EBP implementation), Sympathy (disposition towards EBP)]. Mixed model analysis with sequential Bonferroni adjustment was used to analyse the matched data. Effect sizes (ES) (95% CI) were calculated for all changes.

**Results:**

Effect sizes of the changes in EBP domains were small (ES range 0.02 to 0.42). While most changes were not significant there was a consistent pattern of decline in scores for Relevance in the first workforce year (ES -0.42 to -0.29) followed by an improvement in the second year (ES +0.27). Scores in Terminology improved (ES +0.19 to +0.26) in each of the first two workforce years, while Practice scores declined (ES -0.23 to -0.19) in the first year and improved minimally in the second year (ES +0.04). Confidence scores improved during the second workforce year (ES +0.27). Scores for Sympathy showed little change.

**Conclusions:**

During the first two years in the workforce, there was a transitory decline in the self-reported practice and sense of relevance of EBP, despite increases in confidence and knowledge. The pattern of progression of EBP skills beyond these early professional working years is unknown.

## Background

In evidence based practice (EBP) there is a growing body of research concerning educational practices, barriers and facilitators, but few studies have tracked longitudinal change in EBP skills, knowledge or attitudes. The primary goal of entry-level training in the health disciplines is to attain competency in a set of skills and knowledge that is an acceptable starting point in a specific health profession. During the immediate years after graduation from entry-level training, professional performance (skills, knowledge and decision-making) is likely to improve as a result of workplace-based experience (exposure to a variety of patients, feedback from peers and senior staff) [1]. Variation has been reported in a number of health professions for specific professional skills during the early years in the workforce [2,3]. This may be due to the initial entry-level training (breadth rather than depth of training, mismatch between entry-level curricula and workplace expectations), the supervision provided in the workplace or access to educational opportunities and resources. While it is likely that all individuals will improve initially with work place experience, only those professionals who engage and continue to engage in deliberate practice of specific skills are likely to continue to improve in performance [1].

Since the publication of the Sicily statement [4], there has been greater attention paid to EBP training in entry-level education (Bachelor degrees or equivalent). This consensus document recommended that every individual practitioner be trained in the five-step model of EBP with skills to ask a research question, access, appraise and apply the evidence, and assess the process. More recently, Glasziou [5] advocated that EBP training should be introduced early, and embedded and applied throughout entry-level training [5]. In theory, this training should 'future-proof' graduates with the life-long learning skills required for making evidence-based health care decisions [4].

The majority of investigations exploring the impact of EBP education have been undertaken in the medical and nursing disciplines [6]. In disciplines outside of medicine and nursing such as physiotherapy and occupational therapy, there are a number of studies reporting the learning outcomes of EBP education in clinicians or post-graduate students [7,8], with fewer studies available for entry-level students [9,10]. After completing a half semester course, significant improvements were reported in research skills (interpretation and application of research), with decreased confidence in research abilities and a sustained strong belief in attitudes to the importance of research [9]. Long et al [10] reported significant improvements in self-reported EBP knowledge, attitudes and behaviours after undertaking two consecutive entry-level EBP courses (ES range 0.30-1.34).

To date, few studies have evaluated the changes in EBP attitudes, knowledge and skills during transition from entry-level training into the professional workplace. Connolly et al. [11] and Sabus [12] evaluated changes in EBP domains in 34 and 31 physiotherapists after twelve and three months in the workforce respectively. Connolly et al. [11] reported declines in confidence (3%) and the relevance of research (13%) with no change in sympathy (effect sizes not reported). Sabus [12] reported a significant decline in EBP competency (confidence) (ES 0.69) but not attitudes to the use of evidence in practice ('clinical behaviours') (ES 0.01). There has been limited tracking of EBP practice and the understanding of EBP in allied health professionals after graduation.

The aim of this study was to describe changes in EBP knowledge, attitudes and behaviours of physiotherapy graduates during their initial years in the professional workforce.

## Methods

### Overview

An observational, prospective, longitudinal study design with matched data was used. Ethical approval for the study was granted by the Human Research Ethics Committee of the University of the Australia (Ethics protocol P180/08).

### Sample

Participants from two consecutive cohorts (2008, 2009) of entry-level physiotherapy students training at the University of South Australia were recruited. Data were collected at the end of the final year in the entry-level program and reassessed at the end of their first and second years in the workforce. Strategies were implemented to minimise non-participation. These included updating contact details, reminder emails six months prior to questionnaire delivery and follow-up emails to non-responders.

Within the entry-level program, training in EBP was undertaken via formal stand-alone courses, and integrated in a number of professional theoretical courses and supervised clinical practice. The EBP research evidence competencies taught and assessed in the entry-level physiotherapy program at the University of South Australia are summarised in Table 1 (adapted from Lewis 2010) [13]. The EBP content was essentially similar across both the 2008 and 2009 cohorts.

**Table 1:** Summary of research evidence competencies in courses undertaken by study participants (adapted from Lewis 2010) [13]

	Research evidence competency						
**Course**	**Research question**	**Search strategy**	**Research design**	**Critical appraisal knowledge**	**Critical appraisal application**	**Hierarchy of evidence knowledge**	**Statistics**

1	**C A**	**C A**	**C A**	**C A**	**C A**	**C A**	**C A**

2		**C A**				**C A**	

3	**C A**	**C**	**C A**	**C**	**C**	**C**	**C A**

4	**A**	**A**				**A**	

5	**C**	**C**	**C A**	**C A**	**C**		**C A**

6	**C A**	**A**	**C A**			**A**	**C A**

7	**C**	**C**	**C**	**C**			

8	**C A**	**C A**	**C A**	**C A**	**C A**	**C A**	**C A**

Cells marked with a 'C' indicate that the research evidence competency was included in the course curriculum. Cells marked with an 'A' indicate that the competency was specifically assessed.

### Instrument

The Evidence-Based Practice Profile Questionnaire (EBP^2^) [14] was used in the study. The questionnaire included 74 items all of which used a 5-point Likert scale. Eleven demographic items are included (age, profession, employment, EBP training and highest qualification achieved). The first 58 items related to one of five domains of EBP (Relevance, Terminology, Confidence, Practice, and Sympathy). A definition of these domains, the number of items included in the domain after factor analysis and examples of domain items are presented in Table 2. There were 16 additional non-domain items that described environmental and personal characteristics that might act as barriers or facilitators to EBP. These items were not included in the analysis. A full copy of the EBP^2 ^questionnaire and a scoring sheet are provided as additional files with this submission (Additional files 1 and 2). Individual domain item scores were aggregated to give a domain score (5-point Likert scale, minimum score of 1 and a maximum score of 5 per item). A combined overview of these domains may be used to develop an EBP 'profile'. Space was provided in the questionnaire for the addition of comments. The questionnaire required 10-12 minutes to complete. The EBP^2 ^had been previously evaluated for reliability and validity (test retest ICCs 0.77-0.94, convergent validity r = 0.52-0.80, discriminative validity ANOVA *p <*0.001 to 0.004) [14]. Questionnaires were prepared and delivered using commercially available web-based survey software (SurveyMonkey™).

**Table 2:** Overview of domains within the EBP^2 ^instrument including illustrative examples of items

Domain	Definition	Item Numbers	Examples of items
Relevance	Relevance refers to the value, emphasis or importance placed on EBP	1-14 (14 items)	Rate your response to the following statements: EBP helps me make decisions about clients in my work Application of EBP is necessary in my work

Terminology	Terminology refers to an understanding of common research and statistical terms;	22-38 (17 items)	Rate your understanding of the following terms: Odds ratio Confidence interval

Sympathy*	Sympathy refers to the compatibility of EBP with professional work	15-21 (7 items)	Rate your response to the following statements: EBP does not take into account the limitations of my day-to-day work EBP does not take into account my clients' preferences

Practice	Practice refers to the use of EBP in clinical situations	39-47 (9 items)	In the past year how often have you: Considered your clients' preferences when making clinical/professional decisions Integrated research evidence with your expertise

Confidence	Confidence refers to a perception of ability with EBP skills	48-58 (11 items)	Rate your confidence in the following EBP activities: Ability to search an electronic database Ability to determine how useful (clinically applicable) the material is

*for scoring, items 15-21 need to have Likert score reversed: 1-5, 2-4, 3-3, 4-2, 5-1

### Data management and data analysis

Due to the multiple dependent variables (domain scores), alpha was set at 1%, rather than 5%, to minimise the likelihood of a significant result occurring by chance. The target power was set at 80%. The sample size was calculated to detect a medium effect size, if such an effect existed. A Cohen's f of 0.25 (equivalent to a Cohen's d of 0.5) was set as the target [15]. The minimum sample size required to detect such an effect size was 40 across three test occasions and 51 across two test occasions.

Questionnaire data were imported into SPSS Statistics 17.0 (Chicago, IL). Data were checked for accuracy and cleaned. Descriptive statistics were calculated for demographic data. Missing values were imputed for all items that were to be collated into a domain score. Since the data were categorical, multiple imputation was used but allowing for the data to be whole numbers [16]. Domain scores were calculated for each participant for the five EBP domains, for each time the questionnaire was completed.

A random coefficient regression or mixed model analysis was used to analyse matched data (same participant on each occasion of follow-up) for the five domain scores, compared across three test occasions for the 2008 cohort (final entry-level year, first and second year in the workforce) and across two test occasions (final entry-level year and first year in the workforce) for the 2009 cohort. Alpha was set at 5%; however due to the multiple comparisons (five EBP domains) a sequential Bonferroni adjustment was used to limit the risk of a Type 1 error [17]. Pairwise post-hoc comparisons used Bonferroni correction. Effect sizes (Cohen's d, 95% CI) were calculated for all changes. Contemporary interpretations of effect sizes based on Thalheimer and Cook (2002), adapted from Cohen (1988) were used [negligible (≥ 0 and < 0.15), small (≥ 0.15 and < 0.40), medium (≥ 0.40 and < 0.75), large (≥ 0.75 and < 1.10), very large (≥ 1.10 and < 1.45) and huge (≥ 1.45) [15,18].

## Results

### Participant flow through the study

Recruitment for the study commenced in December 2008 and data collection was completed in December 2010. Due to a delay in finalising the questionnaire, the first data collection for the 2008 cohort commenced in May 2009 (rather than in December 2008, when entry-level training was actually completed). There were 72 students completing entry-level physiotherapy programs in 2008. Of these, 45 gave permission in December 2008 to be contacted. In May 2009 32 were able to be contacted and data was analysed from 29 who completed all three questionnaires (in the final entry-level year and after the first and second years in the workforce). In the 2009 cohort, 90 out of 96 participants completed the testing as students in their final entry-level year. In December 2010, there were 80 of these participants eligible for inclusion after a year in the workforce. The ten ineligible participants graduated later than the remainder of their cohort, due to leave of absence or failure in a course during the program, and had not competed a full year in the workforce by December 2010. Of the 80, who were eligible, four participants did not complete the second questionnaire (two were unable to be contacted, two did not respond to the invitation). Matched data from 29 subjects (2008 cohort) over three test occasions and 76 (2009 cohort) over two test occasions were analysed. Data were analysed separately for each of the two cohorts because of the potential for confounding secular changes influencing the analysis, due to differences in timeframes of testing.

On the first occasion of questionnaire administration, the 2008 (n = 29) and 2009 (n = 76) cohorts were similar for age (2008: mean age 24.2 ± 4.2 years, range 21-40; 2009 mean age 23.8 ± 4.6 years, range 20-44) and gender proportion (females to male in 2008: 2.6:1, in 2009: 1.8:1). The non-participants in the 2008 cohort were of similar age (mean age 23.2 ± 3.0 years, range 21-35) with a higher female to male ratio (1.5:1).

### 2008 cohort

There were significant (*p *< 0.05) main effects for time for Terminology (*p *= 0.01), Confidence (*p *= 0.02) and Practice (*p *= 0.04), but these did not reach significance with sequential Bonferroni adjustment. There were no significant (*p *< 0.05) main effects for time for Relevance (*p *= 0.25) and Sympathy (*p *= 0.26). The sample size for the 2008 cohort (n = 29) did not reach the required estimate based on the power analysis (n = 40). This part of the study was therefore underpowered. Pairwise post-hoc comparisons were not undertaken due the absence of significant findings.

Table 3 summarises the descriptive data for the five domain scores [raw mean scores, SD, CI and the effect sizes (95%CI)] for changes between occasions of testing for the 2008 cohort. The effect sizes have been calculated based on the adjusted mean (SD) for random effects mixed modelling.

**Table 3:** Descriptive data and analyses for the 2008 cohort (n = 29) across the five EBP domains.

Domain	Range^	Final year mean (SD) 95% CI	1^st ^year workforce mean (SD) 95% CI	2^nd ^year workforce mean (SD) 95% CI	Final →1^st ^year workforce *ES 95% CI	1^st ^→ 2^nd ^year workforce *ES 95% CI	Final → 2^nd ^year workforce *ES 95% CI
Relevance	14-70	60.2 (5.5) 58.1-62.3	59.0 (5.5) 56.9-61.1	60.0 (5.5) 57.9-62.1	ES -0.29 -0.81 to +0.23	ES +0.27 -0.23 to +0.77	ES -0.04 -0.55 to +0.48

Terminology	17-85	60.0 (8.3) 56.8-63.1	61.5 (9.9) 57.7-65.2	63.2 (10.1) 59.4-67.0	ES +0.19 -0.32 to +0.71	ES +0.22 -0.29 to +0.73	ES +0.42 -0.10 to +0.94

Confidence	11-55	40.0 (6.8) 37.5-42.6	39.7 (8.5) 36.4-42.9	42.5 (6.9) 39.8-45.1	ES -0.02 -0.50 to +0.55	ES +0.27 -0.25 to +0.79	ES +0.29 -0.23 to +0.90

Practice	9-45	26.6 (6.7) 24.1-29.2	24.9 (5.6) 22.8-27.0	25.0 (6.4) 22.5-27.4	ES -0.23 -0.75 to +0.29	ES +0.04 -0.48 to +0.55	ES -0.27 -0.78 to +0.25

Sympathy	7-35	22.6 (3.7) 21.1-24.0	22.3 (3.9) 20.8-23.8	23.0 (3.0) 21.9-24.2	ES -0.15 -0.66 to +0.37	ES +0.22 -0.30 to +0.74	ES +0.07 -0.59 to +0.44

CI confidence interval; ES effect size. ^ a higher score indicates improvement

*ES calculated based on adjusted mean (SD) for random effects mixed modelling;

→ change from period to period.

Compared to scores achieved as final year students, Practice scores declined after one year (*p *= 0.04, ES -0.23) and remained lower after two years in the workforce (*p *= 0.02, ES -0.27). Compared to scores achieved as final year students, negligible change in Confidence scores (*p *= 0.88, ES -0.02) was found at the end of the first year in the workforce with a greater increase between the first and second year in the workforce (*p *= 0.02, ES +0.27). Both Confidence and Terminology scores were higher (*p *= 0.01, ES +0.29, *p *= 0.004, ES +0.42 respectively) after two years in the workforce than as final year students. There were minor changes with small effect sizes in scores for Relevance and Sympathy across the three test occasions (Table 3).

### 2009 cohort

Significant main effects for time for Relevance (*p *= 0.0002) and Terminology (*p *= 0.004) were found and these remained significant after sequential Bonferroni adjustment. No significant (*p *< 0.05) main effects for time for Practice (*p *= 0.06), Confidence (*p *= 0.14) and Sympathy (*p *= 0.58) were found. This part of the study was adequately powered to detect a medium ES.

Table 4 summarises the descriptive data for the five domain scores [raw mean scores, SD, CI and the effect sizes (95% CI)] of changes for the 2009 cohort. The effect sizes have been calculated based on the adjusted mean (SD) for random effects mixed modelling.

**Table 4:** Descriptive data and analyses for the 2009 cohort (n = 76) across the five domains

Domain	Final year mean (SD) 95% CI	1^st ^year workforce mean (SD) 95% CI	Final year→1^st ^year workforce *ES 95% CI
Relevance	60.7 (5.6) 59.4-62.0	57.5 (6.1) 56.1-58.9	**ES -0.42** **+0.74 to +0.09**

Terminology	58.0 (8.8) 56.0-60.0	60.5 (10.1) 58.2-62.8	**ES +0.26** **+0.05 to +0.47**

Confidence	40.6 (6.1) 39.2-42.0	40.8 (7.2) 39.1-42.4	ES +0.14 -0.18 to +0.45

Practice	26.1 (6.7) 24.5-27.6	24.0 (5.3) 22.8-25.2	ES -0.19 -0.51 to +0.13

Sympathy	22.1 (3.4) 21.3-22.9	21.2 (3.7) 20.4-22.0	ES -0.06 -0.38 to +0.26

CI confidence interval; ES effect size; Bolded results indicate significant findings (p < 0.05) after Bonferroni adjustment; *ES calculated based on adjusted mean (SD) for random effects mixed modelling; → change from period to period.

Compared to scores achieved as final year students, after one year in the workforce, Relevance scores declined significantly (*p *= 0.0002, ES -0.42) while scores for Terminology significantly improved (*p *= 0.004, ES +0.26). While not significant (*p *> 0.05) across the first two years in the workforce compared to scores achieved as final year students While not significant (*p *> 0.05) across the first two years in the workforce compared to scores achieved as final year students, Practice (ES -0.19) and Sympathy (ES -0.06) scores declined and an improvement was found in Confidence (ES +0.14) scores (Table 4).

### Patterns of change

**Figure 1:**
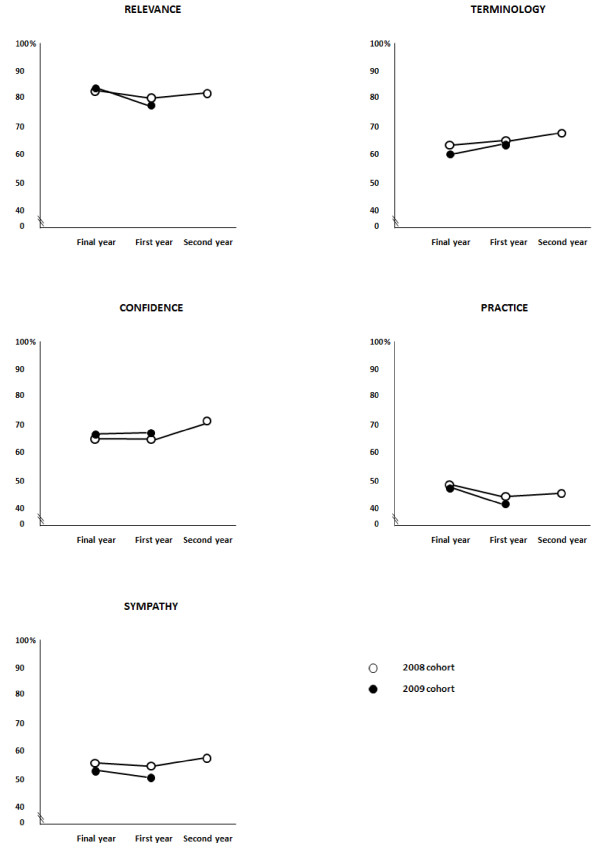
**Scores (percentage maximum) for Relevance, Terminology, Confidence, Practice, Sympathy for the 2008 and 2009 cohorts**.

Mean scores as a percentage of the maximum score for each domain of Relevance, Terminology, Confidence, Practice and Sympathy for both cohorts are illustrated in Figure 1. While statistical significance varied, the patterns were clear and consistent across the cohorts.

## Discussion

### Key findings

In the current study, changes in self-reported EBP domains were evident during the transition from the final year of entry-level training into the early workforce years. This was the first study of an allied health professional discipline (physiotherapy) to measure changes in five EBP profile domains across the entry-level training-workforce transition for up to two years after graduation. The effect sizes of the changes were small. While few of the changes were statistically significant, there were consistent patterns of change. In particular, Relevance declined with transition into the workforce, then increased during the second year; Terminology continued to improve and Confidence increased during the second year. Practice declined after a year in the workforce and there was little further change in the second year. Sympathy scores changed only minimally.

### Progression of EBP skills

Using the medical profession as an example, Ericsson [1] described progressive changes in clinical skills after graduation. On entry into the workforce, clinical skills initially improved as a result of increased frequency of skills performance or exposure to practice and feedback provided by external sources (supervisors, colleagues, patients). However, after initial improvement, these then began to decline over a number of years. New health care graduates move into workplaces where they will generally be supported by clinically-skilled peers (in varying stages of 'ascent' and 'decline'). In contrast, during the immediate years in the workforce, health professional graduates are likely to be exposed to greater variance, modelling and exposure to EBP skills than professional clinical skills. The level of peer skill, attitude and practice to support early EBP integration is less certain. Without ongoing practice and support, a quicker demise in EBP compared with clinical skills would not be surprising.

### Influences on the 2008 and 2009 cohorts

The introduction of EBP training in entry-level education based upon the recommendations of the Sicily statement is relatively recent. The two cohorts involved in this longitudinal study commenced their entry-level programs in 2004 (2008 cohort) and 2005 (2009 cohort) which were the year prior to, and the year of, the publication of the Sicily statement [4]. These two cohorts were therefore likely to be involved in the early stages of introduction of formal EBP training in the physiotherapy program. Components of EBP were interspersed through other courses but the influence of these may have depended on the experience and exposure of the person conducting the teaching. Participants in the study have therefore undertaken their degrees while EBP training has been in a stage of development rather than having highly developed EBP concepts and teaching throughout the program.

### The EBP domains

#### Terminology

Terminology related to the understanding of 17 terms, such as 'confidence interval', 'odds ratio' and 'systematic review'. The findings suggested overall improvements in the understanding of the terminology used in EBP in the first and second years after graduation with a medium effect size after two years of working compared to graduation. Previous systematic reviews involving mostly medical students and graduates [4,19] have reported that knowledge is a characteristic of EBP which has been shown to improve with EBP training. The self-reported knowledge domain of EBP may follow a similar pattern to clinical skills, with an increase as a result of greater exposure in the early years after graduation. It may also be possible that new graduates perceive they have increasing knowledge about EBP terminology because they are comparing themselves with workplace colleagues demonstrating variable levels of EBP knowledge and behaviours.

#### Relevance

Relevance reflected an individual's attitudes toward EBP, intentions to develop knowledge and skills and the value placed on EBP in decision-making. Attitudes have been a characteristic that is considered difficult to teach [4] and may be dependent on the working culture and environment. The entry-level program completed by all participants included education, application and assessment of EBP. At the end of their entry-level training participants appeared to value EBP (83% maximum score). If "attitudes are caught not taught" [4], the decline in Relevance after one year may reflect attitudes in the workplace environment which may not consistently embed, prioritise or provide opportunities to practise EBP. A similar decline in scores for the relevance of research was also reported by Connolly et al. (2001) at one year after graduation.

#### Confidence

Confidence related to self-perceived abilities in EBP skills. The 11 items addressed question formulation, access, appraisal, application of EBP and research and computer skills. It was interesting to observe that significant changes in Confidence scores occurred only after two years and were not apparent after a year in the workforce. The negligible change in Confidence scores during the first year of working may be a result of adjusting to a range of professional work and lifestyle changes. In the second year in the workforce, as a more settled routine is established, graduates may have become more comfortable with the workplace, leading to small improvements (ES 0.29) in Confidence scores. The time lapse before the change also supports the value of longer term follow-up and suggests that not only are changes in EBP small, but in some domains the changes take longer to occur. While the items in this domain are clearly directed at Confidence in *EBP skills*, it may be that the increase in Confidence scores, reflected greater confidence in all areas of professional skills rather than separating EBP-specific from clinical skills. While minimal changes in confidence were also reported by Connolly et al. (2001) after working for a year, the marked decline in the study by Sabus (2008) may be due to the shorter timeframe (three months) between graduation and re-evaluation.

#### Practice

Practice pertained to the application of EBP skills in relation to clinical work. The items in this domain required the participant to indicate how frequently (never to daily) they engaged in EBP behaviours such as formulating a question, accessing and appraising evidence, integration with expertise and patient preferences in decision-making and sharing of findings with others in the workplace and profession. Practice scores declined after a year of working and remained lower than pre-graduation scores after two years in the workforce. The pattern of these changes differs to the expected progression of clinical skill changes after graduation [1] and may reflect the absence of peer/workplace resources to support for EBP skills. These findings are concerning as they seem in contrast to reported improvements in Confidence and Terminology; that is, participants were not practising their EBP skills but reported greater Confidence in their EBP skills and their understanding of EBP-related Terminology.

#### Sympathy

The Sympathy items related to the compatibility of EBP in the workplace and in the profession. Specific items included limitations the workplace may impose on the practice of EBP, and balancing client preferences, clinical experience and availability of high level evidence. Scores for Sympathy were relatively low and changed little across all test occasions. In the current study, scores for Sympathy were low and remained relatively unchanged (range 55-57% maximum score) from the final year of training across the first two years of working. The findings suggest that participants were not entering environments conducive to the ongoing development of EBP skills. In addition, the practitioners with whom they work are also likely to vary in their support and empathy for EBP. Alternatively, as the inclusion of formal EBP training in entry-level physiotherapy curricula was only commenced during the time these participants were students, the overall academic environment may have been equally as diverse in terms of EBP support as the work environment into which the participants transitioned.

### Implications of this study

The pattern of findings in the current study imply that, at the time of data collection, participants may have found little opportunity to exploit their EBP skills in the workforce. This suggests that there is a need to consider how EBP "training" can be extended both in entry-level education and into the workplace. This is in line with recommendations by Glasziou et al. [5] for a 'multi-pronged' approach to embedding EBP skills into health professions, with catch-up programs for those already qualified and development of infrastructure to support and facilitate skills and integration of EBP.

### Limitations

While the sample size for the 2009 cohort (n = 76) exceeded the estimate required, the sample size for the 2008 cohort was smaller than expected (n = 29) and failed to reach the number required to detect medium effect sizes (80% power, alpha 0.01). However, the pattern of findings is consistent across the 2008 and 2009 cohorts (Figure 1). There may be less confidence though in accepting the absence of significant differences for the 2008 cohort from the first to the second year in the workforce as there was no comparative pattern to observe in the 2009 cohort.

While data from 29 of 32 participants was included across three occasions of testing, the initial response rate from the 2008 cohort was low. Consent was able to be gained from 45 of a potential 72 graduates as they completed their studies in November 2008. Final year students have varied timelines for final courses and locations of these and may have less opportunity to access and respond to email in this busy period. Many chose not to attend end-of-year social functions where recruitment occurred. Of the 45 students who gave consent, 32 were able to be contacted when the questionnaire was completed in May 2009. The remaining 13 did not return the questionnaire or were not able to be contacted (change of/incorrect email address, moves from student residence).

The self-report questionnaire (EBP^2^) used in this study had been previously evaluated for reliability and validity [14]. However, the nature of all self-report questionnaires is that they capture perceived, rather than true or actual measures. Differences in interpretation of items may remain a consideration but should have less impact due to the use of repeated measures from the participants. With further developments in embedding EBP training in entry-level programs, the challenge will be to ensure further clarification of EBP concepts to enhance the certainty with which participants complete the questionnaire.

The participants were drawn from an accessible population (physiotherapy students in a metropolitan university) which limits the generalisability of the study results. Respondent bias cannot be disregarded as participants were graduates of the University and may have responded in accordance with their satisfaction with entry-level training. The bias was considered to be reduced by a statement in the information sheet regarding responses having no impact on the participant's future relationship with the university, by encouragement for honesty in answers and because the research team had no influence on the ongoing employment of these graduates.

## Conclusions

The current study was the first longitudinal study, using a questionnaire which had demonstrated reliability and validity, to monitor the change in attitudes, knowledge and behaviours of physiotherapists as they transitioned from entry-level training into the workforce. The findings suggested that participants, all of whom had been exposed to entry-level EBP training, graduated with a strong perception of the importance of EBP, were confident and had a good understanding of EBP terminology but engaged in little practice of EBP. In the first year in the workforce there was a general decline in practice and the value placed on EBP but improvements in understanding of common terminology. In the group of participants assessed at the end of their second year in the workforce, there were improvements in confidence and understanding of EBP terminology with little evidence of changes in EBP-related practice. It is encouraging that there were significant changes, and that EBP teaching appeared to "work". Furthermore, the fact that changes in the perceived understanding of terminology and in confidence were independent of changes in sympathy and practice, may say something about the need to include some EBP "attitudinal training", emphasising perhaps the philosophical importance of EBP.

The pattern of change in these EBP domains beyond the first two years in the workforce remains unknown. With the inconsistency in EBP exposure of workplace colleagues, a decline in EBP-specific skills could be expected. Alternatively, with increased numbers of EBP-trained graduates entering the workforce in the future, there may be changes in workplace culture, support and resources which will raise EBP skill development opportunities leading to stronger, more positive EBP profiles. The findings of this study suggest the need for an extension of EBP training into the workplace, as the ultimate test of any educational training (entry-level or workplace) will be the demonstration of evidence-based clinical management decisions and positive changes in patient outcomes.

## Competing interests

The authors declare that they have no competing interests.

## Authors' contributions

MPM, MTW, TSO, LKL all instigated and conceived of the study, participated in the design of the study, participated in development and testing of the questionnaire and edited the manuscript. MPM co-ordinated the data collection. JP performed the statistical analysis. All authors read and approved the final version.

## Pre-publication history

The pre-publication history for this paper can be accessed here:

http://www.biomedcentral.com/1472-6920/11/100/prepub

## Supplementary Material

Additional file 1 **Evidence-Based Practice Profile (EBP^2^) questionnaire.pdf is a copy of the complete questionnaire used in this study**. Click here for file

Additional file 2 **Scoring for the Evidence-Based Practice Profile questionnaire outlines the scoring for the domain items in the Evidence-Based Practice Profile questionnaire**. Click here for file

## Author information

Maureen McEvoy DipTech(Physio), MAppSc(Physio), PhD

Lecturer Physiotherapy, School of Health Sciences, University of South Australia

Marie Williams BAppSc(Physio), PhD

Associate Professor, School of Health Sciences, University of South Australia.

Tim Olds BA(Hon), BSpSc, PhD(Syd), PhD(UNSW)

Professor of Health Sciences, University of South Australia

Lucy Lewis BAppSc(ExSpSc), BPhysio, BHSc(Hons), PhD

Lecturer Physiotherapy, School of Health Sciences, University of South Australia

John Petkov BSc, BAppSc, MSc

Senior Lecturer, University of South Australia

## References

[B1] Ericsson KA Deliberate practice and the acquisition and maintenance of expert performance in medicine and related domains Acad Med 2004 79 10 S70 S80 15383395 10.1097/00001888-200410001-00022

[B2] Fromme HB Karani R Downing SM Direct observation in medical education: a review of the literature and evidence for validity Mt Sinai J Med 2009 76 365 371 10.1002/msj.20123 19642150

[B3] Pfeiffer C Madray H Ardolino A Willms J The rise and fall of student's skill in obtaining a medical history Med Ed 1998 32 283 288 10.1046/j.1365-2923.1998.00222.x 9743783

[B4] Dawes M Summerskill W Glasziou P Cartabellotta A Martin J Hopayian K Porzsolt F Burls A Osborne J Sicily statement on evidence-based practice BMC Med Ed 2005 5 1 10.1186/1472-6920-5-1 PMC544887 15634359

[B5] Glasziou P Burls A Gilbert R Evidence based medicine and the medical curriculum. The search engine is now as essential as the stethoscope BMJ 2008 337 a1253 a704 a705 18815165 10.1136/bmj.a1253

[B6] Flores-Mateo G Argimon JM Evidence based practice in postgraduate healthcare education: A systematic review BMC Health Serv Res 2007 7 119 10.1186/1472-6963-7-119 PMC1995214 17655743

[B7] McCluskey A Lovarini M Providing education on evidence-based practice improved knowledge but did not change behaviour: A before and after study BMC Med Ed 2005 5 40 10.1186/1472-6920-5-40 PMC1352357 16364181

[B8] Stevenson K Lewis M Hay E Do physiotherapists' attitudes towards evidence-based practice change as a result of an evidence-based educational programme? J Eval Clin Pract 2004 10 2 207 217 10.1111/j.1365-2753.2003.00479.x 15189387

[B9] Heiss DG Basso DM Physical therapy on trial: The rationale, organization, and impact of a mock trial on physical therapy students' attitudes toward and confidence in research J Allied Health 2003 32 3 202 210 14526904

[B10] Long K McEvoy M Lewis L Wiles L Williams M Olds T Entry-level evidence-based practice (EBP) training in physiotherapy students - Does it change knowledge, attitudes and behaviours? A longitudinal study IJAHSP 2011 9 3

[B11] Connolly BH Lupinnaci NS Bush AJ Changes in attitudes and perceptions about research in physical therapy among professional physical therapist students and new graduates Phys Ther 2001 81 5 1127 1134 11319938

[B12] Sabus C The effects of modeling evidence-based practice during the clinical internship J Phys Ther Ed 2008 2 3 74 84

[B13] Lewis L Evidence based practice in entry-level physiotherapy education PhD thesis 2010 University of South Australia, School of Health Sciences

[B14] McEvoy MP Williams MT Olds TS Development and psychometric testing of a trans-professional evidence-based practice profile questionnaire Med Teach 2010 32 9 e373 e380 10.3109/0142159X.2010.494741 20795796

[B15] Cohen J Ed Statistical Power Analysis for the Behavioural Sciences 1988 2 Hillsdale, New Jersey: Lawrence Erlbaum Associates 397

[B16] Vermunt JK van Ginkel JR van der Ark LA Sijtsma K Multiple imputation of incomplete categorical data using latent class analysis Sociol Methodol 2008 38 1 369 397

[B17] Holm S Simple sequentially rejective multiple test procedure Scand J Stat 1979 6 2 65 70

[B18] Thalheimer W Cook S How to calculate effect sizes from published research articles: a simplified methodology 2002 http://education.gsu.edu/coshima/EPRS8530/Effect_Sizes_pdf4.pdf accessed February 18, 2011

[B19] Coomerasamy A Khan KS What is the evidence that postgraduate teaching of evidence based medicine changes anything? A systematic review BMJ 2004 329 7473 1017 10.1136/bmj.329.7473.1017 15514348 PMC524555

