# Supplementary material for: Evidence-based practice profiles of physiotherapists transitioning into the workforce: a study of two cohorts
Source: BMC Med Educ. 2011 Nov 29;11:100. doi: 10.1186/1472-6920-11-100 (PMC3248363; doi:10.1186/1472-6920-11-100)
Supplement: Additional file 1 — Evidence-Based Practice Profile (EBP2) questionnaire.pdf is a copy of the complete questionnaire used in this study. [file 1472-6920-11-100-S1.PDF]

UNIVERSITY OF SOUTH AUSTRALIA

Evidence-Based Practice Profile Questionnaire

The aim of this questionnaire is to collect data on evidence-based practice (EBP) knowledge, behaviours and attitudes

Survey instructions

We would be very appreciative if you could please take some time to complete this survey.
It will take 10-12 minutes to complete.
Please circle one number in each line or tick/answer as requested.
Comment on your responses as appropriate in the areas provided

Thank you for your time in completing this questionnaire

Rate your RESPONSE to the following statements:

	Not at all true	Not really true	Possibly true	Quite likely true	Very true
1. I understand what is meant by the term evidence-based practice (EBP)	1	2	3	4	5
2. I am aware of EBP in my profession	1	2	3	4	5
3. My profession uses EBP as a framework	1	2	3	4	5
4. I am aware of current developments in EBP in my profession	1	2	3	4	5

Do you have any comments about your responses?

Rate your RESPONSE to the following statements:

	No intention at all	Unlikely to consider doing it	Could consider doing it	Highly likely to consider doing it	Absolutely intend to do it/keep doing it
5. I intend to develop knowledge about EBP	1	2	3	4	5
6. I intend to develop skills in accessing, acquiring and appraising evidence relevant to my area of practice	1	2	3	4	5
7. I intend to read relevant literature to update knowledge	1	2	3	4	5
8. I intend to apply best available evidence findings to improve practice	1	2	3	4	5

Do you have any comments about your responses?

Rate your RESPONSE to the following statements:

	Strongly Disagree	Disagree	Neutral	Agree	Strongly agree
9. Application of EBP is necessary in my work	1	2	3	4	5
10. Literature and research findings are useful in my day-to-day work	1	2	3	4	5
11. I need to increase the use of evidence in my daily work	1	2	3	4	5
12. I am interested in learning or improving the skills necessary to incorporate EBP into my work	1	2	3	4	5
13. EBP improves the quality of my work	1	2	3	4	5
14. EBP helps me make decisions about clients in my work	1	2	3	4	5
15. EBP does not take into account the limitations of my day-to-day work	1	2	3	4	5
16. There isn't much point in doing EBP because there is a lack of strong evidence to support most of the work I do	1	2	3	4	5
17. EBP does not take into account my clients' preferences	1	2	3	4	5
18. In making decisions about my professional work, I value clinical/field experience more than scientific studies	1	2	3	4	5
19. Workplace experience is the most reliable way to know what really works	1	2	3	4	5
20. Critical appraisal of the literature and its relevance to the client is not very practical in the real world of my profession	1	2	3	4	5
21. Seeking relevant evidence from scientific studies is not very practical in the real world	1	2	3	4	5

Do you have any comments about your responses?

Rate your UNDERSTANDING of the following terms:

	Never heard the term	Have heard it but don't understand	Have some understanding	Understand quite well	Understand and could explain to others
22. Relative risk	1	2	3	4	5
23. Absolute risk	1	2	3	4	5
24. Systematic review	1	2	3	4	5
25. Odds ratio	1	2	3	4	5
26. Meta analysis	1	2	3	4	5
27. Number needed to treat	1	2	3	4	5
28. Confidence interval	1	2	3	4	5
29. Publication bias	1	2	3	4	5
30. Forest plot	1	2	3	4	5
31. Intention to treat	1	2	3	4	5
32. Statistical significance	1	2	3	4	5
33. Minimum clinically worthwhile effect	1	2	3	4	5
34. Clinical importance	1	2	3	4	5
35. Randomised controlled trial (RCT)	1	2	3	4	5
36. Dichotomous outcomes	1	2	3	4	5
37. Continuous outcomes	1	2	3	4	5
38. Treatment effect size	1	2	3	4	5

<u>Do you have any comments about your responses?</u>

IN THE PAST YEAR HOW OFTEN have you:

	Never	Monthly or less	Fortnightly	Weekly	Daily
39. Formulated a clearly answerable question that defines the client or problem, the intervention and outcome(s) of interest	1	2	3	4	5
40. Tracked down the relevant evidence once you have formulated the question	1	2	3	4	5
41. Searched an electronic database	1	2	3	4	5
42. Critically appraised any literature you have discovered to determine the methodological quality	1	2	3	4	5
43. Integrated research evidence with your expertise	1	2	3	4	5
44. Considered your clients' preferences when making clinical/professional decisions	1	2	3	4	5
45. Read published research reports	1	2	3	4	5
46. Informally shared and discussed literature/research findings with others in your workplace	1	2	3	4	5
47. Formally shared and discussed literature/research findings with others in your department/practice (eg journal club, in-service presentation)	1	2	3	4	5

<u>Do you have any comments about your responses?</u>

Rate your CONFIDENCE in the following EBP activities:

	Not at all confident	A little confident	Reasonably confident	Quite confident	Very confident
48. Research Skills	1	2	3	4	5
49. Computer skills	1	2	3	4	5
50. Ability to identify gaps in your knowledge	1	2	3	4	5
51. Ability to convert your information needs into clearly answerable questions	1	2	3	4	5
52. Awareness of major information types and sources	1	2	3	4	5
53. Ability to search an electronic database	1	2	3	4	5
54. Ability to access evidence (get copies of articles or reports)	1	2	3	4	5
55. Ability to critically analyse evidence against set standards ie quality scoring	1	2	3	4	5
56. Ability to determine how valid (close to the truth) the material is	1	2	3	4	5
57. Ability to determine how useful (clinically applicable) the material is	1	2	3	4	5
58. Ability to apply information to individual cases (ie integrate research evidence with personal preferences, values, concerns, expectations)	1	2	3	4	5

Do you have any comments about your responses?

Rate your RESPONSE to the following statements:

	Strongly Disagree	Disagree	Neutral	Agree	Strongly Agree
59. I want to learn new information	1	2	3	4	5
60. I critically evaluate new ideas	1	2	3	4	5
61. I have good management skills	1	2	3	4	5
62. I solve problems using a plan	1	2	3	4	5
63. I enjoy studying	1	2	3	4	5
64. In my organisation, leaders continually look for opportunities to learn	1	2	3	4	5
65. I make time to read research	1	2	3	4	5
66. Insufficient time is one of the greatest barriers to the use of EBP in my clinical/professional practice	1	2	3	4	5
67. My workload is too great for me to keep up to date with all the new evidence	1	2	3	4	5
68. The cost of information resources limits my use of EBP in my clinical/professional practice	1	2	3	4	5
69. Easy access to computers dictates whether or not I practise EBP	1	2	3	4	5
70. The resources available to me are adequate to undertake EBP	1	2	3	4	5
71. Collective support amongst my colleagues is one of the greatest facilitators to my use of EBP in clinical/professional practice	1	2	3	4	5
72. Support from management is one of the greatest facilitators to my use of EBP in clinical/professional practice	1	2	3	4	5
73. Senior management/my employer requires me to use EBP	1	2	3	4	5
74. I've just had a gutful of EBP	1	2	3	4	5

<u>Do you have any comments about your responses?</u>

Demographics

75. What is your age? ______
76. When is/was your final year as an undergraduate student?_________
77. Sex: ☐ Female ☐ Male
78. Are you currently working in the profession for which you have trained/are training?
    ☐ YES ☐ F/T ☐ P/T
    ☐ NO
79. What is that profession?______________________

80. Which of the following best describes your MAIN work setting: *(Tick one box only)*
    ☐ Public sector ☐ Private sector ☐ Academic
    ☐ Community-based agency (Domiciliary Care, Community Centre, Charitable institution)
    ☐ Other (please specify) ____________________________

81. Type of work: In which area have you mainly worked in the past year?
(Tick one box only)
    ☐ Managers (eg Health/Education Manager)
    ☐ Education (eg teacher or educator at University, School)
    ☐ Health (eg Diagnostic, Promotion, Therapy, Allied Health, Medical, Nursing)
    ☐ Information, Communication, Technology
    ☐ Legal, Social and Welfare
    ☐ Arts and Media
    ☐ Business, Human Resources and Marketing
    ☐ Design, Engineering, Science and Transport
    ☐ Other (please specify) ____________________

82. Please provide a brief overview of your work experience in the past 12 months:
____

83. Are you currently studying? ☐ NO
    ☐ YES ☐ F/T ☐ P/T
84. If YES, what are you studying? ____________________________

85. What is your highest qualification attained? *(Tick one box only)*

☐ Registered Nurse	☐ Diploma
☐ Post graduate certificate course	☐ Graduate Diploma
☐ Bachelor	☐ Honours (Bachelor WITH Honours <u>or</u> Bachelor AND Honours degree)
☐ Masters course work /Graduate Entry	
☐ Masters (Research)	☐ PhD
☐ Other (Please specify) _____________	

86. Have you formally undertaken any training in EBP? ☐ NO ☐ YES
    If YES: choose longest completed course if you have done more than one
    ☐ EBP course as part of University education (Bachelor, Masters etc) >20 hrs
    ☐ Short course 10 - 20 hours
    ☐ Weekend course 3 - 10 hrs
    ☐ Single lecture 1- 3 hrs
87. Is English your first language? ☐ YES ☐ NO
