# Supplementary material for: Evidence-based practice profiles of physiotherapists transitioning into the workforce: a study of two cohorts
Source: BMC Med Educ. 2011 Nov 29;11:100. doi: 10.1186/1472-6920-11-100 (PMC3248363; doi:10.1186/1472-6920-11-100)
Supplement: Additional file 2 — Scoring for the Evidence-Based Practice Profile questionnaire outlines the scoring for the domain items in the Evidence-Based Practice Profile questionnaire. [file 1472-6920-11-100-S2.DOC]

**Scoring for the Evidence-Based Practice Profile (EBP^2^) Questionnaire**

McEvoy MP, Williams, MT, Olds TS (2010) Development and psychometric testing of a trans-professional evidence-based practice profile questionnaire. *Medical Teacher* 32(9):e366-73.

The EBP^2^ questionnaire has 87 items and takes under 12 minutes to complete.

Factor analysis identified 5 domains which incorporate the first 58 items.
There are an additional 16 non-domain items.
There are 13 demographic items.

| **DOMAINS/SECTIONS** | **ITEM NUMBERS** | **SCORING** (min-max) |
| --- | --- | --- |
| Relevance | 1-14 (14 items) | 14-70 |
| Sympathy* | 15-21 (7 items) | 7-35 |
| Terminology | 22-38 (17 items) | 17-85 |
| Practice | 39-47 (9 items) | 9-45 |
| Confidence | 48-58 (11 items) | 11-55 |
| Non-domain items | 59-74 (16 items) | No scoring |
| Demographics | 75-87 (13 items) | Not applicable |

**for scoring,* *items 15-21 need to have Likert score reversed: 1-5, 2-4, 3-3, 4-2, 5-1*
